# Fetal Aberrant Subclavian Artery (ARSA): flow dynamics and its impact on fetal development assessed by doppler ultrasonography

**DOI:** 10.61622/rbgo/2026rbgo5

**Published:** 2026-02-20

**Authors:** Melih Bestel, Elif Ucar, Salim Sezer

**Affiliations:** 1 Istanbul Esenyurt University Faculty of Health Sciences Department of Midwifery Istanbul Turkey Department of Midwifery, Faculty of Health Sciences, Istanbul Esenyurt University, Istanbul, Turkey.

**Keywords:** Aberrant Right Subclavian Artery (ARSA), Vascular anomaly, Fetal development, Anatomical variation, Ultrasound parameters, Congenital anomaly

## Abstract

**Objective:**

This study aimed to evaluate the hemodynamic characteristics of fetuses with isolated Aberran Right Subclavian Artery (ARSA) and compare them with a control group of normal fetuses using Doppler ultrasonography.

**Methods:**

A total of 93 fetuses were included in this prospective case-control study. Forty fetuses with isolated ARSA and 53 control fetuses with normal right subclavian arteries were analyzed. Doppler ultrasonographic parameters (Peak Systolic Velocity (PS), Pulsatility Index (PI), Resistive Index (RI), Time-Averaged Maximum Velocity (TAMAX), Heart Rate (HR)) were assessed and compared between the two groups.

**Results:**

No significant differences were found in the Doppler parameters (PS, PI, RI, TAMAX, HR) between the ARSA and control groups. Furthermore, there was no correlation between gestational age, fetal weight, and Doppler parameters in either group.

**Conclusion:**

The study supports the hypothesis that isolated ARSA is a benign anatomical variant without significant hemodynamic impact on fetal development. ARSA cases without associated cardiac or chromosomal anomalies do not appear to affect fetal growth.

## Introduction

Aberrant right subclavian artery (ARSA) is one of the most common congenital anomalies of the aortic arch. Although often asymptomatic, it can sometimes be associated with other anatomical or chromosomal abnormalities.^(1)^ In the general population, ARSA occurs in approximately 0.5% to 4.4% of live births, most often diagnosed incidentally.^(2-4)^

Its incidence in chromosomally normal fetuses ranges between 0.4% and 1.5%.^(5,6)^ In the general population, the aortic arch typically gives rise to three major vessels: the brachiocephalic artery, the left common carotid artery, and the left subclavian artery. However, in the presence of ARSA, the aortic arch gives rise to four vessels: the right common carotid artery, the left common carotid artery, the left subclavian artery, and the right subclavian artery (also known as arteria lusoria).

Embryologically, ARSA results from the abnormal regression of the right dorsal aorta. It commonly courses posterior to the esophagus, where it may compress the trachea, leading to symptoms such as dyspnea, cough, and dysphagia.^(7,8)^ Less frequently, it has been associated with retrosternal pain, significant weight loss exceeding 10 kg over six months, abdominal pain, back pain, and numbness in the right upper extremity.^(8)^ However, ARSA is generally asymptomatic.^(9)^

**Figure 1:**
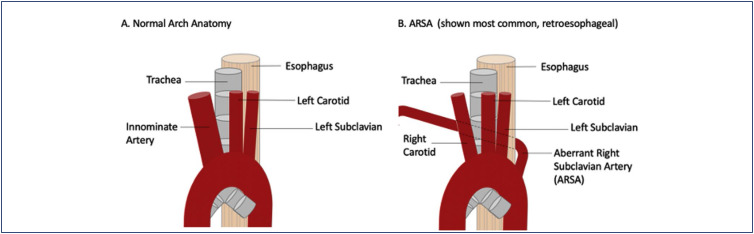
Normal Arch and ARSA Anatomy ^(10)^

In cases of isolated ARSA without additional pathologies, studies have shown no significant issues with growth or development after birth.^(4)^ Consequently, it can often be considered a normal anatomical variant (Figure 1).^(10)^

ARSA - Aberrant Subclavian Artery

ARSA has been associated with certain chromosomal abnormalities, most notably Trisomy 21 (Down syndrome), with a reported prenatal prevalence of 6.8%–37.5% in fetuses with Down syndrome.^(11-13)^ Studies have highlighted a significantly increased prevalence of ARSA in fetuses with Down syndrome, suggesting its potential use as an ultrasonographic soft marker for this condition.^(14)^

Additionally, ARSA has been linked to DiGeorge syndrome (22q11.2 microdeletion) and other rare genetic disorders.^(11)^ One study found that pathological chromosomal variations occurred in 4.2% of isolated ARSA cases and in 9.4% of cases where ARSA was accompanied by other anomalies.^(4)^

Research indicates that while isolated ARSA is generally benign, its presence alongside other ultrasonographic abnormalities increases the risk of chromosomal anomalies. Prenatal genetic evaluation (e.g., karyotyping, SNP analysis, or cffDNA testing) can aid in understanding and managing such cases.^(11)^

Fetuses with isolated ARSA are typically asymptomatic both during fetal life and postnatally. However, as discussed above, a small proportion may experience symptoms. The unique origin of ARSA from the aortic arch, its aberrant course, and the potential for tracheal compression suggest that alterations in blood flow velocity, pulsatility, and resistance could occur in this artery.

To date, no Doppler studies examining ARSA have been reported in the literature. To test this hypothesis and evaluate the potential clinical implications, we designed this prospective Doppler study focused on ARSA.

## Methods

**Figure 2:**
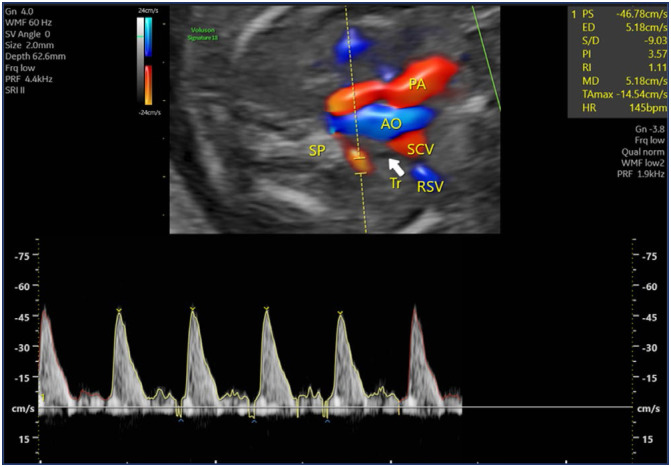
Color Doppler image of the aberrant right subclavian artery (ARSA)

**Figure 3:**
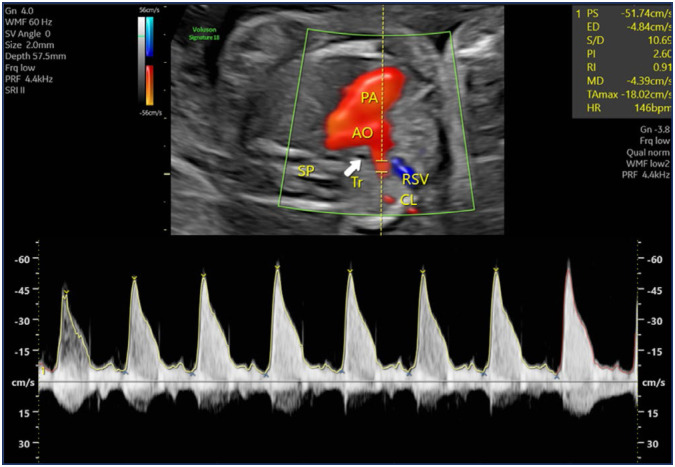
Color Doppler image of a normal right subclavian artery

Our study was conducted as a prospective case-control study between January and December 2023 at a tertiary center where numerous fetal anomalies are managed. The study included fetuses diagnosed with ARSA during second-trimester ultrasounds performed at 20–24 weeks of gestation in the perinatology clinic, as well as fetuses without any anomalies serving as controls. Fetuses with additional pathological findings, including cardiac, extracardiac, or chromosomal anomalies, were excluded from the study. Information on patients diagnosed with ARSA during examinations was recorded, and their details were retrieved from the hospital database. Examinations were performed using a Voluson E8 (GE Healthcare, Austria) device with a Rab 6 probe. Doppler assessments in fetuses with ARSA were conducted by a single experienced perinatologist specializing in Doppler studies. Measurements were taken at the proximal segment of the ARSA immediately after its emergence from the aortic arch, ensuring an insonation angle of less than 30 degrees (Figure 2). In the control group, Doppler measurements were obtained at a similar insonation angle, just after the subclavian artery originated from the aortic arch and before it reached the clavicle (Figure 3).

Color Doppler image of the aberrant right subclavian artery (ARSA) in a fetus at 21 weeks and 5 days of gestation. Note that the ARSA originates from the aortic arch and courses posterior to the trachea (indicated by the arrowhead) and anterior to the spinal vertebra. The Doppler sample gate is placed over the ARSA. PA: Pulmonary artery, AO: Aorta, SVC: Superior Vena Cava, RSV: Right Subclavian Vein, Tr,: Trachea; SP: Spinal Vertebra

Color Doppler image of a normal right subclavian artery in a fetus at 22 weeks of gestation. The right subclavian artery is seen coursing anterior to the trachea (indicated by the arrowhead) and extending toward the area beneath the clavicle. The Doppler sample gate is placed on the subclavian artery just after its origin from the aorta and before it reaches the clavicle. PA: Pulmonary artery, AO: Aorta, SVC: Superior Vena Cava, RSV: Right Subclavian Vein, Tr,: Trachea; SP: Spinal Vertebra, CL: Clavicle

Statistical analyses were performed using the SPSS software package (IBM SPSS Statistics 27). Frequency tables and descriptive statistics were used to interpret the findings. For measurement values following a normal distribution, parametric methods were applied. To compare measurement values between two independent groups, the "Independent Sample-t" test (t-test) was used. For values not following a normal distribution, non-parametric methods were used. The "Mann-Whitney U" test (Z-test) was applied to compare measurement values between two independent groups. The "Spearman" correlation coefficient was used to examine the relationships between two quantitative variables that did not follow a normal distribution.

The study was approved by the local review board and the ethics committee (protocol no:2022/02-8) Before participation, all individuals were verbally informed, and written consent was obtained. All research procedures complied with the 1964 Helsinki Declaration and its later amendments or ethical standards.

## Results

In our cohort, 40 fetuses were diagnosed with ARSA, and none of them were found to have chromosomal abnormalities, detailed information regarding chromosomal assessment:

- 7 patients had undergone non-invasive prenatal testing (NIPT) prior to the ARSA diagnosis.
- 6 patients opted for amniocentesis after ARSA was detected.
- 11 patients underwent NIPT following the ARSA diagnosis.
- The remaining 16 patients declined further genetic testing, citing financial constraints or personal/religious reasons.

A total of 93 fetuses were included in the study, comprising 40 fetuses with isolated ARSA and 53 control fetuses without pathological findings. There were no statistically significant differences in the demographic data between the groups (p > 0.05) (Table 1).

**Table 1:** Comparison of demographic findings according to groups

Variable	ARSA (n=40)		Control (n=53)		Statistical analysis* Probability
		Median [IQR]		Median [IQR]	
Age(years)	31.10±4.55	30.5 [6.0]	30.38±5.64	30.0 [8.0]	t=0.663 p=0.509
Gravida	2.02±1.09	2.0 [2.0]	2.09±1.21	2.0 [1.5]	Z=-0.131 p=0.896
Parity	0.68±0.85	0.0 [1.0]	0.78±0.78	1.0 [1.0]	Z=-0.838 p=0.402
Abortion	0.35±0.62	0.0 [1.0]	0.32±0.78	0.0 [0.0]	Z=-1.154 p=0.249
Curettage	0.00±0.00	0.0 [0.0]	0.00±0.00	0.0 [0.0]	Z=0.000 p=1.000
Gestational week	22.64±2.42	22.4 [2.1]	22.68±2.02	22.1 [2.5]	Z=-0.481 p=0.630
Fetal weight (gr.)	576.20±299.33	502.5 [255.8]	584.52±213.95	544.0 [227.0]	Z=-1.145 p=0.252

* ‘Independent Sample’ test (t-table value) statistics were used to compare the measurement values of two independent groups for normally distributed data. ‘Mann-Whitney U’ test (Z-table value) statistics were used to compare the measurement values of two independent groups for the data that do not have normal distribution

No statistically significant differences were observed between the groups regarding PS, PI, RI, TAMAX, and HR values (p > 0.05) (Table 2).

**Table 2:** Comparison of some quantitative findings by groups

Variable	ARSA (n=40)		Control (n=53)		Statistical analysis* Probability
		Median [IQR]		Median [IQR]	
PS	-7.74±49.08	-39.0 [94.9]	9.91±57.36	46.5 [112.9]	Z=-1.536 p=01.24
PI	4.62±1.99	3.9 [1.8]	4.55±1.51	4.1 [2.3]	Z=-0.233 p=0.816
RI	1.08±0.07	1.1 [0.1]	1.06±0.09	1.1 [0.1]	Z=-0.754 p=0.451
TAMAX	-2.52±12.81	-10.0 [23.5]	1.40±15.19	7.8 [29.8]	Z=-0.640 p=0.522
HR	146.90±12.34	148.5 [13.0]	150.38±6.94	149.0 [9.0]	Z=-0.855 p=0.392

* Mann-Whitney U’ test (Z-table value) statistics were used in the comparison of two independent groups with measurement values in data that did not have normal distribution

In the ARSA group, no statistically significant correlations were found between gestational age, fetal weight, and PS, PI, RI, TAMAX, and HR values (p > 0.05) (Table 3). Similarly, in the control group, no statistically significant correlations were observed between gestational age, fetal weight, and PS, PI, RI, TAMAX, and HR values (p > 0.05).

**Table 3:** Examination of the relationships between gestational week and fetal weight (gr.) and some quantitative findings by groups

Correlation *		ARSA (n=40)		Control (n=53)	
		Gestational week	*Fetal weight (gr.)*	Gestational week	*Fetal weight (gr.)*
PS	*r* *p*	-0.096 0.558	-0.110 0.500	-0.136 0.331	-0.075 0.595
PI	*r* *p*	-0.057 0.728	-0.033 0.837	0.092 0.513	-0.093 0.506
RI	*r* *p*	-0.020 0.905	0.050 0.706	0.140 0.319	0.153 0.274
TAMAX	*r* *p*	-0.012 0.939	-0.061 0.707	-0.159 0.256	-0.114 0.418
HR	*r* *p*	-0.054 0.741	-0.013 0.937	0.073 0.601	0.058 0.681

* ‘Spearman’ correlation coefficient was used to analyse the relationship between two quantitative variables that do not have normal distribution

## Discussion

The findings of this study provide valuable insights into the hemodynamic characteristics of fetuses with isolated ARSA and address an area not explored in previous Doppler studies. Despite theoretical assumptions about altered blood flow dynamics due to ARSA's unique anatomical origin and abnormal trajectory, our results revealed no significant differences in key Doppler parameters (PS, PI, RI, TAMAX, and HR) between fetuses with ARSA and those in the control group. Since this vessel supplies arterial blood flow to the right arm and no changes were observed in Doppler studies, it suggests that ARSA is unlikely to cause any issues for the fetus, particularly for the right arm, during the prenatal period.

The absence of significant differences supports the hypothesis that isolated ARSA, in the absence of additional anomalies, is typically a benign anatomical variation without hemodynamic implications during fetal life. These findings align with previous studies suggesting that isolated ARSA does not adversely affect fetal growth or postnatal development. Our study also confirms the absence of significant correlations between gestational age, fetal weight, and Doppler parameters in both groups. This consistency emphasizes the stability of ARSA as an isolated finding without broader physiological effects.

Fetal weight is an important parameter in monitoring pregnancy and generally provides insight into the fetus's overall health. There is limited data suggesting that ARSA significantly impacts fetal growth rates. Most studies indicate that isolated ARSA does not have a notable effect on fetal growth.^(15)^

For example, while there are some findings suggesting that conditions such as fetal growth restriction (IUGR) are more frequently observed in fetuses with ARSA, this is generally not seen in isolated cases without accompanying pathological findings. In one study, when comparing the fetal weights of fetuses with ARSA to those without pathological findings, no statistically significant difference was found.

The limited impact of ARSA on fetal development may be due to its recognition as an isolated finding in most cases. There are many factors that determine the relationship between fetal weight and gestational age; however, ARSA does not appear to have a significant effect on these parameters. That being said, fetal weight can be influenced by other factors, such as genetic abnormalities or cardiovascular disorders.^(4)^

Doppler ultrasonography, a method used to assess blood flow, can be used to detect the presence of ARSA; however, these findings are generally not associated with fetal growth or genetic anomaly risks. In this study, no significant difference was observed in Doppler ultrasonographic parameters between fetuses with ARSA and the control group. These findings suggest that when ARSA is considered an isolated finding, it does not directly affect the fetus's cardiovascular development, and as a result, Doppler ultrasonographic parameters in these fetuses continue to remain within normal ranges.^(11)^

Since the ultrasonographic evaluation of ARSA is not routinely performed in the first trimester and ARSA-related fetal aneuploidy cases may have a lower incidence due to spontaneous abortion or pregnancy termination before birth, we believe that the observed incidence is lower than normal.

In cases where ARSA is associated with more complex cardiovascular anomalies, changes in Doppler ultrasonographic parameters may be observed. For example, in more serious cardiovascular anomalies such as ARSA with Kommerell's diverticulum, significant changes in fetal circulation and differences in Doppler measurements may be seen. However, these cases are typically different from the isolated presence of ARSA and require further information regarding the fetus's cardiovascular health.^(16)^

There are numerous publications in the literature regarding arterial malformations. The Adachi classification, which consists of five types, is used for variations of the hypogastric artery.^(17)^

In the variation of the hypogastric artery, the embryonic umbilical artery, which is a regressed vessel of the IIA complex, becomes significant. These vessels and their variations play a crucial role in the vascular supply of visceral organs.^(18)^

Fetal venous system abnormalities are quite rare in prenatal screening and clinically exhibit a variety of manifestations.^(19)^ Abnormalities affecting the umbilicus, hepatic, and portal systems have been observed to not lead to significant clinical outcomes in the fetus. However, abnormalities in the IVC and SVC can have important clinical implications.^(20,21)^

Some of these include cardiomegaly and hydrops fetalis that develop in the antenatal period. Venous defects can affect individual systems, and their combination can also occur, potentially related to abnormalities in the arterial system.^(19)^

The strength of this study lies in the fact that, while many studies have investigated the association of ARSA with chromosomal anomalies, no research has yet evaluated the Doppler findings in isolated ARSA cases. The absence of any associated abnormal Doppler findings allows us to consider this finding as a variant of normal. Although the normal Doppler findings do not necessarily indicate that this vessel is a normal variant, the absence of negative consequences in the organ it supplies supports the hypothesis that it is a benign anatomical variation with no hemodynamic impact during fetal life.

## Conclusion

Isolated ARSA does not have a significant impact on fetal growth. While ARSA rarely leads to clinical issues, it can be associated with swallowing difficulties (dysphagia) or rare complications in some cases. When counseling families with fetuses diagnosed with isolated ARSA, it can be reassuring to inform them that no significant change in blood flow to the right arm has been detected, meaning there will be no weakness in the arm. Further research with larger sample sizes could help confirm these findings and explore long-term potential outcomes.

## Data Availability

the authors did not make the data from this article available in repositories prior to submission.
